# A single-arm phase II study of nab-paclitaxel for patients with chemorefractory non-small cell lung cancer

**DOI:** 10.1186/s12885-017-3684-8

**Published:** 2017-10-16

**Authors:** Hisashi Tanaka, Kageaki Taima, Takeshi Morimoto, Yoshihito Tanaka, Masamichi Itoga, Kunihiko Nakamura, Akihito Hayashi, Mika Kumagai, Hideo Yasugahira, Megumi Mikuniya, Koichi Okudera, Shingo Takanashi, Sadatomo Tasaka

**Affiliations:** 1 Department of Respiratory Medicine, Hirosaki University Graduate School of Medicine, Zaifu-cho 5, Hirosaki, 036-8562 Japan; 2 Department of Respiratory Medicine, Hachinohe City Hospital, Hachinohe, Japan; 3 Department of Respiratory Medicine, Hirosaki Chuo Hospital, Hirosaki, Japan; 4 Health Administration Center, Hirosaki University, Hirosaki, Japan

**Keywords:** Lung cancer, Nab-paclitaxel, Refractory

## Abstract

**Background:**

We aimed to evaluate the efficacy and safety of nab-paclitaxel in patients with refractory advanced non-small cell lung cancer who failed previous chemotherapy.

**Methods:**

Patients were required to have an Eastern Cooperative Oncology Group performance status of 0–2 and adequate organ function. Patients received nab-paclitaxel, 100 mg/m^2^ i.v. on days 1, 8, and 15 every 4 weeks. The primary endpoint was the overall response rate. Secondary endpoints were the progression-free survival time, overall survival, and the toxicity profile.

**Results:**

From July 2013 to July 2015, a total of 31 patients were enrolled. Fourteen patients received nab-paclitaxel as a second-line and 17 received it as an over third-line therapy. Each patient received a median of 5 treatment cycles (range, 1–11). The overall response rate was 19.3% (95% confidence interval, 9.1–36.2%) (complete response (*n* = 0), partial response (*n* = 6), stable disease (*n* = 17), and progressive disease (*n* = 8)). The median progression-free survival time was 4.5 months (95% confidence interval 3.5–6.3 months), median overall survival time was 15.7 months, and 1-year survival rate was 54.8%. Most common grade 3 or 4 non-hematological toxicities were elevated aspartate transaminase level (3.2%) and sensory neuropathy (9.6%). Neutropenia was the most common grade 3 or 4 adverse events (38.6%), and febrile neutropenia developed in 12.9% patients. No treatment-related deaths were observed in this study.

**Conclusion:**

Primary endpoint was met. Single agent nab-paclitaxel showed significant clinical efficacy and manageable toxicities for patients with chemorefractory advanced non-small cell lung cancer even if late line setting.

**Trial registration:**

UMIN000011696. The date of registration was July 11th, 2013.

## Background

Lung cancer is the leading cause of cancer death related to cancer in the world, with non–small cell lung cancer (NSCLC) accounting for 85% of lung cancer cases [1]. For advanced or metastatic NSCLC, platinum-based chemotherapy is the mainstay of first-line treatment [2–4]. In the last decades, encouraging new treatments have afforded benefits to patients with adenocarcinoma. Patients with certain driver oncogene such as epidermal growth factor receptor (EGFR) mutation, anaplastic lymphoma kinase (ALK) fusion, and c-ros oncogene 1 (ROS1) fusion gene are recommended to receive molecular target therapy [5]. Most patients receiving platinum doublet therapy as the first-line however, they experience disease progression and next line therapy. Second-line chemotherapy also has beneficial effects on overall survival. In previous randomized controlled phase III trials, docetaxel, pemetrexed and erlotinib are recognized as standard second-line therapies [6–8]. More recently nivolumab represents a new treatment option for patients requiring second-line treatment for metastatic non-small cell lung cancer [9, 10]. Based on the results of phase III clinical trials, the use of immune checkpoint inhibitors could be the treatment in second-line setting.

Nanoparticle albumin-bound paclitaxel (Nab-PTX) is a paclitaxel (PTX) formulation in which nanoparticles of PTX are bound to human serum albumin. Because this formulation is free of the solvent that is used for the conventional PTX formulation, this formulation can be administered to alcohol-hypersensitive patients. In pre-clinical study, nab-PTX was significantly less toxic than PTX, and nab-PTX is comprised of a colloidal suspension of albumin and PTX which probably enhances drug delivery of the cytotoxic agent to the cancer cells [11]. CA031 was a randomized phase III trial that compared carboplatin plus nab-PTX with carboplatin plus PTX as first line chemotherapy in patients with advanced-stage NSCLC [12]. Nab-PTX arm had a significantly higher overall response rate than PTX arm. However, the efficacy and safety of single agent nab-PTX for chemorefractory patients with advanced NSCLC in Japanese has not been reported yet. In this multicenter phase II study, we aimed to evaluate the efficacy and safety of nab-PTX in patients with chemorefractory advanced NSCLC including an over third-line setting.

## Methods

### Study design

This clinical trial was an open-label, multicenter, single-arm study involving 3 institutions in Aomori, Japan. This study was performed in accordance with the principles of the Declaration of Helsinki and Good Clinical Practice guidelines. This study was approved by the institutional review boards at each institution. Patients selected whether they would participate in this trial after detailed explanation; written informed consent was obtained from all patients before the study entry. This study was registered with the University Hospital Medical Information Network (UMIN). Clinical trial number UMIN000011696.

### Eligibility criteria

Patient eligibility required compliance with the following criteria: histologically or cytologically confirmed NSCLC. The patients were ≧ 20 years, had chemorefractory disease, measurable disease as defined by the Response Evaluation Criteria in Solid Tumors (RECIST) (version 1.1), an Eastern Cooperative Oncology Group (ECOG) performance status (PS) 0–2. Patients also had adequate bone marrow function (peripheral leukocyte count ≧ 3000/mm^3^, neutrophil count ≧ 1500/mm^3^, hemoglobin ≧ 9.0 g/dL, and platelet count ≧ 100,000/mm^3^), an adequate function of other organs includes aspartate transaminase and alanine transaminase levels ≦ 2.0 × the upper limit of normal, creatinine ≦ 1.5 mg/dl, total bilirubin concentration ≦ 1.5 mg/dl, and PaO2 ≧ 60 Torr or SpO2 ≧ 95%. The life expectancy more than 8 weeks was required. Patients who had undergone thoracic radiation therapy were required to finish their last treatment at least 12 weeks prior to registration in the protocol. Patients with symptomatic central nervous system metastasis, uncontrolled pleural effusion, pregnancy or lactation, the use of corticosteroid or immunosuppressive drugs or medical problems such as active peptic ulcer, heart disease, interstitial pneumonia or pulmonary fibrosis, cerebrovascular disease, and diabetes mellitus were excluded.

### Treatment plan

Patients were received nab-PTX, 100 mg/m^2^ i.v. on days 1, 8, and 15 every 4 weeks. Treatment was discontinued when the patients had disease progression, and observed unacceptable toxicity and the patient refused protocol treatment. Restarting was approved when adequate organ function was recovered and fulfilled the following criteria: the neutrophil count was ≧ 1500/mm^3^, the platelet count was ≧ 100,000/mm^3^, total bilirubin was ≦ 1.5 mg/dl, the ECOG PS was ≦ 2, and the grade of any non-hematologic toxicity was ≦ 2, there was no infection. Before administration of nab-paclitaxel on days 8, 15, the neutrophil count ≧ 500/mm^3^ and the platelet count ≧ 50,000/mm^3^ were required. The dose of nab-PTX was reduced to 75 mg/m^2^ in case of leukopenia or neutropenia of grade 4 persisting for ≧ 5 days, thrombocytopenia of grade 4 or requiring platelet transfusion, febrile neutropenia, or non-hematologic toxicity of grade ≧ 3 during the previous courses. Second dose reduction 50 mg/m^2^ was done if these toxicities occurred after the reduction of the dose to 75 mg/m^2^. The third dose reduction was not permitted, and the protocol treatment was finished.

### Evaluation and statistical analysis

The primary endpoint was the overall response rate (ORR). Secondary endpoints were the progression-free survival time (PFS), overall survival (OS), and toxicity profiles. Simon’s two-stage minimax design was chosen to determine the number of patients required for our study. The ORR 20% was set for the target activity level, with 5% as the lowest response rate of interest. The study was designed to have 90% power to accept and a 1-sided level of type I error of 5% significance to reject the hypothesis. If one or more out of 13 patients responded in the first stage, this trial could be continued to the second stage. The estimated accrual number was 27 patients. Allowing 10% of the patients to be ineligible, we planned to enroll 30 patients in the study. If ≧5 responses were observed by the end of the study, we considered that the primary endpoint was met. The PFS time and OS were estimated using the Kaplan–Meier method. The PFS has been defined as the time from the date of the start of treatment to the date of disease progression or death or the date of last contact. If neither event is observed, it is considered to be censored with the latest observation date. If the date on which the exacerbation on the image has been confirmed has exceeded 8 weeks since the last examination date, it shall be censored with the previous examination date. If post-treatment is started, it is considered to be censored with the treatment start date. If the event is unknown because it is a transfer or a non-arrival, it will be terminated with the date of the final survival confirmation. The OS time has been defined as the time from the date of the start of treatment to the date of death or last contact. In patients who cannot follow up, they are censored on the day that survival is confirmed before becoming impossible to pursue. Statistical analyses were performed using JMP 10 (SAS Institute, Cary, NC, USA). Tumor responses were assessed using chest radiography, computed tomography scan at every cycle until disease progression. Unidirectional measurements were adopted on the basis of the RECIST, version 1.1. Toxicity was graded according to the National Cancer Institute-Common Toxicity Criteria, version 4.0.

## Results

### Patient characteristics

From July 2013 to July 2015, a total of 31 patients were enrolled from 3 participating institutions in Aomori. Table 1 showed the characteristics of the 31 eligible patients. There were 24 male (77.4%) patients and 7 female (22.6%) patients, with a median age of 66 years (range, 48–81 years). All patients included in this study were Asian. Most patients (87.1%) had a good ECOG PS score of 0–1. The most common histology was adenocarcinoma (51.6%), followed by 12 squamous cell carcinoma (38.7%), non-small cell carcinoma not otherwise specified (NOS) (9.7%). Fourteen patients (45.1%) received nab-paclitaxel as a second-line therapy and 17 patients (54.9%) received it as an over third-line therapy. Only 3 patients (9.6%) were positive and 28 patients (90.4%) were negative or unknown for the EGFR mutation.

**Table 1:** Patient characteristics (*N* = 31)

	Number of patients	%
Sex		
Male	24	77.4
Female	7	22.6
ECOG PS		
0–1	27	87.1
2	4	12.9
Clinical Stage		
IIIB	10	32.2
IV	11	35.6
Recurrence	10	32.2
Histological type		
Adenocarcinoma	16	51.6
Squamous cell carcinoma	12	38.7
Not specified	3	9.7
Smoking history		
Smoker	25	80.6
Non-smoker	6	19.4
No. of prior treatment regimen		
1	14	45.1
2	7	22.5
3 or more	10	32.4

Abbreviations: *ECOG* Eastern Cooperative Oncology Group, *PS* performance status

### Efficacy

**Fig. 1:**
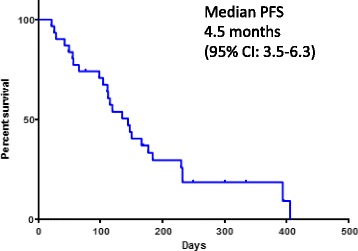
Kaplan–Meier analysis of progression-free survival for all 31 treated patients

**Fig. 2:**
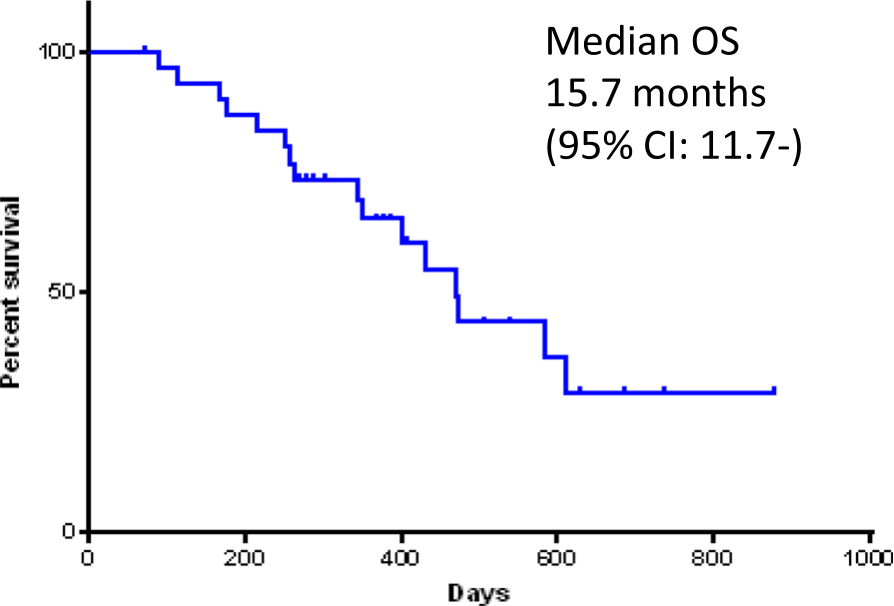
Kaplan–Meier analysis of overall survival for all 31 treated patients

Thirty-one patients were deemed eligible for evaluation of treatment response. Six patients attained a partial response (PR), and no patients attained a complete response (CR). The ORR was 19.3% (95% confidence interval: CI, 9.1%–36.2%), (90% CI, 10.3%–33.2%) (Table 2). Seventeen patients (54.8%) had stable disease (SD) a disease control ratio (DCR) was 74.1%. Eight patients (25.8%) had progressive disease. By the time of analysis, 26 patients had the disease progression events. The OS events occurred in 15 patients. The median PFS was 4.5 months (95% CI, 3.5–6.3 months) (Fig. 1), and the median OS was 15.7 months (95% CI, 11.7 months, not reached) (Fig. 2). The one-year survival rate was 54.8%. Clinical data of post-study treatment were available in 25 patients (80.6%). Twenty-one patients (84.0%) received salvage chemotherapy regimens as post-study treatment. Nine patients in the 1 prior line group received post-study treatment, 3 patients in the 2 prior lines group received post-study treatment and 9 patients in the 3 or more lines group received post-study treatment. Nineteen patients were treated with single agent cytotoxic drug. The three most common agents were vinorelbine (42.0%), S-1 (31.0%) and gemcitabine (21.0%). Two patients with known driver genes were treated with molecular target agents.

**Table 2:** Response to nab-paclitaxel in the intent-to-treat population

Response	Number of patients	%
Complete response	0	0
Partial response	6	19.3
Stable disease	17	54.8
Progressive disease	8	25.9
Response Rate	19.3% (95% CI, 9.1%–36.2%) (90% CI, 10.3%–33.2%)	
Disease control rate	25	74.1

*CI* confidence interval

### Toxicity analysis

The median number of treatment cycles was 5 (range, 1–11 cycles). Fifteen patients (48%) required dose reduction. The primary reasons for dose reduction were grade 4 neutropenia, febrile neutropenia, and grade 3 anemia or neuropathy.

The major toxicities are showed in Table 3. Grade 3 and higher hematologic toxicities included leukopenia (22.5%), neutropenia (38.6%), anemia (3.2%), and thrombocytopenia (0%). No patients received a packed red blood cell transfusion. Febrile neutropenia were observed in 4 patients (12.9%). Grade 3 or 4 non-hematologic toxicities were nausea or vomiting (6.4%), infection (12.9%), sensory neuropathy (9.6%), anorexia (3.2%), and liver dysfunction (3.2%). Most non-hematologic toxicities were generally mild and reversible. No treatment-related deaths were founded in this study.

**Table 3:** Toxicity in patients treated with nab-paclitaxel (*N* = 31)

Toxicity	Grade1/2	%	Grade3	%	Grade 4	%	Grade3/4	%
Leukopenia	21	67.7	6	19.3	1	3.2	7	22.5
Neutropenia	17	54.8	6	19.3	6	19.3	12	38.6
Anemia	25	80.6	1	3.2	0	0	1	3.2
Thrombocytopenia	5	16.1	0	0	0	0	0	0
Febrile neutropenia							4	12.9
Nausea/vomiting	5	16.1	2	6.4	0	0	2	6.4
Anorexia	10	32.2	1	3.2	0	0	1	3.2
Infection	7	22.5	3	9.6	1	3.2	4	12.9
Neuropathy	19	61.2	3	9.6	0	0	3	9.6
Fatigue	22	70.9	0	0	0	0	0	0
Liver dysfunction	10	32.2	1	3.2	0	0	1	3.2
Diarrhea	6	19.3	0	0	0	0	0	0
Hyperkalemia	7	22.5	0	0	0	0	0	0
Edema	0	0	1	3.2	0	0	0	0

## Discussion

This is the first prospective phase II study to evaluate the efficacy and the safety of nab-PTX for patients with previously treated advanced NSCLC including an over third-line setting in Japan. The primary endpoint was ORR. In the present study, the ORR was 19.3%, which is higher than that in previous phase III clinical trials [6–8]. In second-line setting, the ORRs of docetaxel, pemetrexed and erlotinib were reported as 8.2–9.1%, and the median PFSs were 2.2–2.9 months [6–8]. In a phase I-II trial, which evaluated nab-PTX monotherapy as a first-line treatment for NSCLC, the ORR was 30% (12 of 40; 95% CI, 16% to 44%), median PFS was 5.0 months (95% CI, 3 to 8 months), and the 1-year OS was 41% [13]. In another single arm phase II trial, which evaluated nab-PTX monotherapy in a second-line setting, the ORR was 16.1% (9 of 56) and median PFS was 3.5 months (95% CI, 1.9 to 5.8 months), and the 1-year OS was 25% [14]. Liu and colleagues reported a randomized phase II trial comparing nab-PTX (at 150 mg/m^2^ on days 1 and 8 every 3 weeks) with pemetrexed (at 500 mg/m^2^ on day 1 every 3 weeks) in patients with chemorefractory NSCLC. The ORRs were 14.5% in the nab-PTX arm and 10.7% in the pemetrexed arm [15]. The PFS were 5.1 months in the nab-PTX arm and 4.6 months in the pemetrexed arm [15]. In our study, ORR in the both arms were higher than in these previous trials, and PFS was similar. In Western populations, Saxena and colleagues retrospectively evaluated the efficacy of nab-PTX in advanced NSCLC patients with relapsed or chemorefractory disease [16]. They revealed that the ORR was 16.1% and PFS was 3.5 months, which were similar those in previous trials [14, 15]. It was indicated that the efficacy of nab-PTX we observed was better than that in Western populations.

A histology-specific benefit of nab-PTX in patients with advanced NSCLC has been noted [12, 17]. In particular, there was a significant advantage in patients with squamous cell histology. In our study, however, there were no differences in PFS between patients with squamous cell carcinoma and those with other histology (4.3 months versus 5.2 months, *p* = 0.64). It remains to be determined whether the efficacy of nab-PTX is associated with histology.

Our study included the patients who received the treatment as a third or fourth-line. A subgroup analysis revealed that ORR was not different between the second-line setting and over the third-line setting (21.1% versus 17.6%, *p* = 0.79). Nab-PTX was effective even if it was administered as the further line treatments. There have been few prospective studies that indicate the role of over third-line therapy, and they are primarily retrospective analyses. Harada and coworker reported a prospective phase II trial, which evaluated amrubicin monotherapy in third-line or forth-line setting [18]. They showed that the ORR was 9.8% (4 of 41), median PFS was 3.0 months (95% CI, 1.8 to 3.8 months), and the 1-year OS was 53.7% [18]. Both ORR and PFS observed in the present study were superior to the numbers described in the previous report although the 1-year OS was similar [18]. The major limitation in our study is that the sample size might be too small to compare the efficacy of nab-PTX between the second-line and the third-line or later settings. In third-line or forth-line setting, large scale clinical trial is needed to confirm the efficacy of chemotherapy such as nab-PTX or amrubicin monotherapy.

In our study, median OS was 15.7 months which was better than in the previous phase III or phase II trials [6–8]. In phase III trials, the median OS of docetaxel, pemetrexed and erlotinib monotherapy were ranging from 6.8 to 8.3 months [6–8]. In phase II trials, the median OS of nab-PTX were between 6.8 months and 9.8 months [14, 16]. The possible reasons are as follows. Firstly, our study included more stage IIIB (32.2%) and less stage IV patients compared to the previous investigations. Secondly, most patients (84.0%) received subsequent chemotherapy regimens as post-study treatment. The survival outcome might have been influenced by the initial health status of the patients. Furthermore, a selection bias or relatively small sample size might have influenced the data.

## Conclusion

In the present study, nab-PTX is well-tolerated and has significant efficacy in patients with relapsed and previously treated NSCLC even in the third-line or later setting. Obviously, further study is needed. Now phase III clinical trial comparing nab-PTX with docetaxel in patients with previously treated advanced NSCLC is ongoing in Japan. (UMIN00017487).

## Abbreviations

ALK: Anaplastic lymphoma kinase
CI: Confidence interval
CR: Complete response
DCR: Disease control ratio
ECOG: Eastern cooperative oncology group
EGFR: Epidermal growth factor receptor
Nab-PTX: Nanopariticle albmin-bound paclitaxel
NOS: Non-small cell carcinoma not otherwise specified
NSCLC: Non–small cell lung cancer
ORR: overall response rate
OS: Overall survival
PFS: Progression-free survival
PR: Partial response
PS: Performance status
PTX: Paclitaxel
RECIST: Response evaluation criteria in solid tumors
ROS: c-ros oncogene 1
SD: Stable disease
UMIN: University hospital medical information network
